# Modulation of Small RNA Signatures in Schwann-Cell-Derived Extracellular Vesicles by the p75 Neurotrophin Receptor and Sortilin

**DOI:** 10.3390/biomedicines8110450

**Published:** 2020-10-24

**Authors:** Nádia P. Gonçalves, Yan Yan, Maj Ulrichsen, Morten T. Venø, Ebbe T. Poulsen, Jan J. Enghild, Jørgen Kjems, Christian B. Vægter

**Affiliations:** 1 Danish Research Institute of Translational Neuroscience (DANDRITE), Nordic-EMBL Partnership for Molecular Medicine, Department of Biomedicine, Aarhus University, 8000 Aarhus, Denmark; mulr@biomed.au.dk (M.U.); cv@biomed.au.dk (C.B.V.); 2 Interdisciplinary Nanoscience Centre (iNANO), Aarhus University, 8000 Aarhus, Denmark; yanyan@inano.au.dk (Y.Y.); morten.veno@omiics.com (M.T.V.); jk@mbg.au.dk (J.K.); 3 Omiics ApS, 8000 Aarhus, Denmark; 4 Department of Molecular Biology and Genetics, Aarhus University, 8000 Aarhus, Denmark; etp@mbg.au.dk (E.T.P.); jje@mbg.au.dk (J.J.E.)

**Keywords:** exosomes, extracellular vesicles (EVs), miRNAs, Schwann cells, p75^NTR^ receptor, sortilin

## Abstract

Schwann cells (SCs) are the main glial cells of the peripheral nervous system (PNS) and are known to be involved in various pathophysiological processes, such as diabetic neuropathy and nerve regeneration, through neurotrophin signaling. Such glial trophic support to axons, as well as neuronal survival/death signaling, has previously been linked to the p75 neurotrophin receptor (p75^NTR^) and its co-receptor Sortilin. Recently, SC-derived extracellular vesicles (EVs) were shown to be important for axon growth and nerve regeneration, but cargo of these glial cell-derived EVs has not yet been well-characterized. In this study, we aimed to characterize signatures of small RNAs in EVs derived from wild-type (WT) SCs and define differentially expressed small RNAs in EVs derived from SCs with genetic deletions of p75^NTR^ (*Ngfr*^−/−^) or Sortilin (*Sort1*^−/−^). Using RNA sequencing, we identified a total of 366 miRNAs in EVs derived from WT SCs of which the most highly expressed are linked to the regulation of axonogenesis, axon guidance and axon extension, suggesting an involvement of SC EVs in axonal homeostasis. Signaling of SC EVs to non-neuronal cells was also suggested by the presence of several miRNAs important for regulation of the endothelial cell apoptotic process. Ablated p75^NTR^ or sortilin expression in SCs translated into a set of differentially regulated tRNAs and miRNAs, with impact in autophagy and several cellular signaling pathways such as the phosphatidylinositol signaling system. With this work, we identified the global expression profile of small RNAs present in SC-derived EVs and provided evidence for a regulatory function of these vesicles on the homeostasis of other cell types of the PNS. Differentially identified miRNAs can pave the way to a better understanding of p75^NTR^ and sortilin roles regarding PNS homeostasis and disease.

## 1. Introduction

The structural homeostasis is challenging for sensory neurons, whose axons may extend up to more than one meter in humans and with an axonal volume reaching over a thousand times that of the cell body. In a typical mammalian cell, the gene, mRNA, translating machinery and protein destination are a few micrometers apart but the immense size of the axonal compartment challenges the capacity of the neuronal soma to supply and support the constant protein requirements of an entire nerve fiber. A fast microtubule-assisted transport machinery has been identified, conveying material at 20–40 cm/day. However, the capacity of the fast transport is orders of magnitude lower than bulk diffusion (at 1 mm/day), and the cargo seems to be mainly destined for the axon terminals, whereas intrinsic proteins of the axoplasm are not significantly represented [1]. A body of evidence indicates that Schwann cells (SCs) support the axonal maintenance and regenerative responses by diverse mechanisms of cell–cell communication: SCs regulate a wide variety of axonal functions [2], including passive functions associated with myelin formation with subsequent increase in the conduction velocity [3], and more active roles such as enrichment of sodium channels at the nodes of Ranvier [4], specification of the internodal distance [5] as well as metabolic maintenance of the axonal compartment [6]. The regulation of neuronal form and function by SCs has been found to be mediated by different forms of intercellular communication, including coupling via local currents in the periaxonal space, paracrine signaling (e.g., ATP, glutamate) and physical coupling via adhesion molecules and gap junctions [7]. In addition to these classic mechanisms, recent findings suggest the occurrence of lateral molecular cargo transfer mediated by secreted extracellular vesicles (EVs) from SCs to axons [8,9,10]. EVs constitute mainly two types of vesicles: exosomes and microvesicles, which are generated by all cell types and derive from multivesicular bodies or through bubbling of the plasma membrane, respectively [11]. They contain, and are able to transport, proteins, lipids and genetic material such as DNA and RNA with variations in cargo composition depending on, e.g., the age, metabolic state and type of the donor cell [12]. Therefore, in addition to export of cell waste [13], EV-mediated intercellular signaling is an essential component of regulatory neuro-glial communication. In this regard, SC-derived exosomes were found to be internalized by peripheral axons, increasing in vitro neurite sprouting of sensory neurons as well as in vivo axonal regeneration by 50% following nerve crush injury [9,10]. These findings open a new dimension to the intercellular interaction in that the axonal cytoplasm may contain an incomplete translation machinery that is completed by molecules from the SC-derived EVs. The discrete and regular disposition of SCs along the axon may provide full coverage for axonal cargo delivery, suggesting SC-derived EVs participation in the specification of the phenotype of the underlying axon. The current knowledge of SC EVs transcription machinery is, however, very limited. A few recent studies have identified EV components as part of other examinations [9,10,14,15] but a systematic approach to the identification of small RNAs, which by definition are <200 nucleotide in length and usually non-coding RNA molecules, is lacking.

Previous studies have described how the p75 neurotrophin receptor (p75^NTR^) is a key component of the Schwann cell–axon myelination program during development [16] and, depending on binding to neurotrophins or pro-neurotrophins, may mediate cell survival and cytoskeletal remodeling or trigger cell death via p53 and c-Jun N-terminal kinase pathway activation (JNK), respectively [17]. Interestingly, p75^NTR^ was found to be present in SC-derived exosomes, enabling them to lateral transfer to axons [9]. This reveals unforeseen trafficking capabilities of the p75^NTR^ receptor and raises new questions regarding the role of p75^NTR^ for Schwann cell-EV content regulation and signaling. As for p75^NTR^, the Vps10-domain receptor sortilin has also critical roles regarding neurotrophin signaling, as it was shown to be important for regulation of the anterograde axonal transport of Trk receptors and for positively modulating neurotrophin-induced sensory neuronal survival [18]. Sortilin is abundantly expressed in the nervous system during development and adulthood [19], with a perinuclear subcellular localization in intracellular vesicles and the trans-golgi network [20]. When associated with pro-neurotrophins, sortilin forms a complex with p75^NTR^ to induce CNS neuronal apoptosis [21]. In the peripheral nervous system (PNS), sortilin does not seem to be involved in neuron development [18] nor to have a role in sensory neuron apoptosis triggered by a nerve injury [22]. Interestingly, in human lung cancer cells, sortilin was found to be present in exosomes and was further closely linked to the exosomal release mechanism [23]. Therefore, here, we identify small RNA cargos in secreted SC-derived EVs and disclose the paracrine function for p75^NTR^ and sortilin in glial EV production and composition. miRNA signatures identified here might have promising future applications as diagnostic or target tools for PNS-related disease states, which often show compromised neurotrophic signaling as a pathological hallmark.

## 2. Experimental Section

### 2.1. Sprague Dawley Rats

Pregnant wild-type (WT) Sprague Dawley rats were obtained from Janvier labs, while *Ngfr*^−/−^ and *Sort1*^−/−^ Sprague Dawley rats were purchased from Horizon Discovery and custom made, respectively, for breeding in house. *Ngfr*^−/−^ is a deletion within exon 1, across the exon–intron boundary. We have determined that there were no alternative splicing fragments left, proving a clean *Ngfr* deletion. For *Sort1*^−/−^, a 5bp deletion in exon 9 was introduced, resulting in a stop codon, with subsequent completely destruction of the Vps10p-domain folding. No mRNA splice variants or protein fragments were detected by sequencing or Western blot analysis (data not shown).

Rats were housed under a 12 h light/12 h dark cycle in a pathogen-free environment, with water and food ad libitum.

The use of Sprague Dawley rats for obtaining primary Schwann cells was approved by the Danish Animal Experiments Inspectorate under the permission number 2017-15-0201-01192, (with approved date April 2017) and followed the Danish and European animal experimentation legislations (directive 2010/63/EU).

### 2.2. Primary Schwann Cell Culture

Primary Schwann cell cultures were prepared from neonatal (P1–P3) rat pups. In brief, dissected sciatic nerves were digested with 0.25% trypsin (Thermo Fisher Scientific, Waltham, MA, USA), and 0.1% collagenase I (Sigma, St. Louis, MO, USA) in L-15 media (Thermo Fisher), for 30 min at 37 °C. The anti-metabolic agent Cytosine-B-arabino furanoside hydrochloride (Ara-C; 10 μM; Sigma) was used for elimination of fibroblasts, in two cycles of 2–3 days. Purified primary Schwann cells were then expanded with growth media consisting of DMEM (Thermo Fisher) supplemented with 10% FBS (Thermo), 1% penicillin/streptomycin, 2.5 μM forskolin (Sigma) and 10 ng/mL recombinant human neuregulin-1-β1 EGF domain (R&D Systems, Minneapolis, MN, USA) at 37°C in a humidified incubator containing 5% CO_2_.

### 2.3. Immunocytochemistry

Primary Schwann cells were grown in 12 well-plate glass coverslips and fixed with 4% paraformaldehyde for 15 min. After 1 h blocking with 5% donkey serum and 1% bovine serum albumin (BSA) prepared in D-PBS, cells were incubated with rabbit anti-S100 (1:400, Dako, Agilent, Santa Clara, CA, USA), goat anti-sortilin (1:100, R&D Systems) or rabbit anti-p75^NTR^ (1:500, Promega, Madison, WI, USA), diluted in blocking buffer, overnight at 4 °C. Secondary antibodies (Alexa Fluor donkey anti-goat 488 and Alexa Fluor donkey anti-rabbit 568; 1:500, Molecular Probes, Eugenes, OR, USA) were incubated for 2 h at room temperature (RT), together with Hoechst (1:10,000, Sigma) for nuclear staining. Sections were then mounted with Dako Fluorescent mounting medium (Dako) and images acquired with an LSM 780 confocal microscope (Carl Zeiss, Jena, Germany).

### 2.4. Live and Death Assay

The viability of WT, *Ngfr*^−/−^ and *Sort1*^−/−^ primary Schwann cells was analyzed using a combination of calcein-AM (4 μM) and ethidium homodimer-1 (4 μM) (Live/Death Viability/Cytotoxicity Kit, #L3224, Thermo). Primary Schwann cells were seeded into glass coverslips in a 12-well plate (500,000 cells/mL) in DMEM containing 10% FBS and 1% penicillin/streptomycin for 24 h and the assay performed accordingly to the manufacturer’s instructions. The staining was visualized under a fluorescent microscope, with *n* = 6 images per condition (in duplicates) acquired. The number of live (calcein-AM positive) and dead (ethidium homodimer-1 positive) cells were counted and the percentage of live cells (relative to total number of cells) calculated (*n* = 3 independent experiments).

Statistical analysis was accomplished using one-way ANOVA and Tukey’s multiple comparisons post hoc test, with Graph Pad Prism (version 8, San Diego, CA, USA). Quantitative data are reported as mean ± SEM.

### 2.5. Isolation of EVs by Differential Ultracentrifugation

For EV isolation, purified primary Schwann cells were expanded in T175 flasks with growth media. When Schwann cells were about 80% confluent, media was replaced by an identical growth media but containing 10% of an exosome-depleted FBS (Thermo Fisher). After 48 h, media was collected for isolation of the extracellular vesicles (EVs). Two T175 flasks were used per condition and results represent *n* = 8–10 independent experiments. EVs were isolated from the supernatants of Schwann cells by differential ultracentrifugation. Large dead cells and cell debris were eliminated by successive centrifugations at 300× *g* for 10 min, 2000× *g* for 10 min and 10,000× *g* for 30 min, at 4 °C. Finally, supernatants were ultracentrifuged at 100,000× *g* for 120 min, at 4 °C, in a fixed angle rotor (Optima L-80-XP ultracentrifuge, 60 Ti rotor, Beckman-Coulter, Brea, CA, USA) to pellet the EVs. These were then resuspended in 250 μL PBS and immediately stored at −80 °C.

### 2.6. NanoSight Nanoparticle Tracking (NTA) Analysis

The Nanosight LM10 (Malvern Instruments, Malvern, UK) with a 405 nm laser was used to analyze the EVs. The resuspended EVs were diluted 1:100 in PBS and, subsequently, NTA measurements were performed in triplicates, with a 30 s video capture of each sample acquired using camera level set at 16 and detection threshold at 10. NTA software version 3.1 (Malvern Instruments) was used to analyze the data and extract the concentration and size of EVs in the samples.

### 2.7. Western Blotting

WT, *Sort1*^−/−^ and *Ngfr*^−/−^ primary Schwann cells and SC-derived EVs were lysed in standard ice-cold Tris-NaCI-EDTA lysis buffer (supplemented with protease and phosphatase inhibitors) and centrifuged at 10,000× *g* for 20 min at 4 °C. The supernatant was collected, and total protein concentration determined with the Bicinchoninic acid assay (Sigma). Fifty micrograms of total protein samples were separated on 4–12% Bis-Tris protein gels (Thermo Fisher) and electroblotted with nitrocellulose iBlot Gel Transfer Stacks (Invitrogen, Waltham, MA, USA) using the iBlot DryBlotting System (Invitrogen), according to manufactures guidelines. After 1 h blocking with tris buffer saline containing 2% skimmed milk and 2% Tween-20, membranes were incubated overnight at 4 °C with primary antibodies against Sortilin (1:250; R&D Systems) and p75^NTR^ (1:500; Abcam, Cambridge, UK). For the Western blot with the EV pellets, 15 μg of total protein was used and primary antibodies consisted of CD81 (1:100; Santa Cruz Biotechnology, Dallas, TX, USA), as an exosome marker, EEA1 (1:1000; Cell Signaling, Danvers, MA, USA) as a marker for early endosomes and Sortilin (1:250; R&D Systems).

### 2.8. Liquid Chromatography-Mass Spectrometry (LC-MS/MS) Analysis of the EV Pellet

In-solution digest—The exosome sample was lyophilized and resuspended in 8 M urea in 0.1 M ammonium bicarbonate (Ambiec; pH 8.0). The sample was then reduced with 5 mM dithiothreitol (DTT) for 30 min and subsequently alkylated with 15 mM iodoacetamide in for another 30 min, while kept dark. The sample was diluted 5 times in 50 mM Ambic (pH 8.0) and digested overnight with 0.5 µg sequencing grade modified trypsin (Promega, Madison, WI, USA) at 37 °C. The digested sample was micro-purified using Octadecyl C18 Empore extractions disk (3M, St Paul, MN, USA) packed in P10 pipet tips. The purified peptides were suspended in 0.1% formic acid and stored at −20 °C until LC–MS/MS analysis.

LC–MS/MS analysis—The mass spectrometry analysis was performed on an Eksigent nanoLC 415 system (SCIEX, Framingham, MA, USA) connected to a TripleTOF 6600 mass spectrometer (SCIEX) equipped with a NanoSpray III source (SCIEX) and operated under Analyst TF 1.6.0 control (SCIEX). The sample was injected and trapped on a 2 cm in-house packed trap column (id = 100 μm) using RP ReproSil-Pur C18-AQ 3 μm resin (Dr. Maisch GmbH). Peptides were eluted from the trap column and separated on a 15 cm analytical column (id = 75 μm) pulled and packed in-house with RP ReproSil-Pur C18-AQ 3 μm resin (Dr. Maisch GmbH). Peptides were eluted from the column with a flow rate of 250 nL min^−1^, using a 30 min gradient going from 5% to 35% buffer B (acetonitrile with 0.1% formic acid) and sprayed directly into the mass spectrometer. The analysis relied on an information-dependent acquisition method collecting up to 25 MS/MS spectra in each 1.6 s cycle using an exclusion window of 6 s.

Data processing—Raw data obtained from the LC-MS/MS analysis were searched against the Swiss-prot and Trembl databases using the taxonomy rattus (2020_4; SwisProt: 8120 sequences; Trembl: 29,586 sequences) in Mascot 2.5.1 (Matrix science, London, UK). Trypsin was specified as digestion enzyme and allowing 1 miss cleavage. Carbamidomethyl modification of cysteines and oxidation of methionine were selected as fixed and variable modifications, respectively. Mass tolerance of the precursor and product ions was specified as 10 ppm and 0.2 Da using ESI-QUAD-TOF as the instrument setting. The significance threshold was set to 0.01 and the expected cut-off value to 0.005. The search result was imported to MS Data Miner version 1.3.0 (Sourceforge, San Diego, CA, USA) for further processing. Only proteins identified with at least two unique peptides with an ion score of 30 or above were reported as protein identifications (Table S1).

### 2.9. Small RNA Library Preparation and Sequencing

Total RNA from the 250 μL purified EVs was isolated using miRNeasy Serum/Plasma Advanced kit (Qiagen, Hilden, Germany) following the manufacture’s protocol (*n* = 8–10 independent experiments, including different donors). The small RNA library was constructed using 5 μL of the eluted RNA following the manufacture’s protocol of QIAseq miRNA Library Kit (Qiagen). Briefly, 3′ and 5′ adapters were ligated to the RNA. Then the ligated RNAs were reverse transcribed to cDNA using the primer containing the UMI (unique molecule index). Finally, the sample index was introduced by amplification of the library and 22 PCR cycles were used. The quality of the library was checked on the Bioanalyzer using high sensitivity DNA analysis kit (Agilent, Santa Clara, CA, USA) and the quantity was measured using Kapa library quantification kit (Roche, Basel, Switzerland). The libraries were pooled and sequenced using single-end 75 bp sequencing on a Nextseq 500 sequencing machine (Illumina, San Diego, CA, USA).

### 2.10. Small RNA Data Analysis

The raw data were quality filtered and trimmed by fastx_toolkit, and adaptor sequences were removed using Cutadapt. Quality control was performed using FastQC to ensure high quality data. Filtered reads were first mapped to rat tRNA sequences using Bowtie allowing 1 mismatch. Non-mapping reads were then mapped to miRNA sequences using Bowtie allowing 0 mismatches, though allowing the addition of A and T nucleotides at the 3′ end, since miRNAs often have untemplated A and U additions. Reads not mapping to miRNAs were mapped to other relevant transcriptomes (mRNA, rRNA and other small RNAs) and then to the rat (rn6) genome. The expression analysis was done both for miRNA and tRNA. The miRNA and tRNA mapping reads were deduplicated, meaning identical reads with identical UMIs were collapsed to a single read. Then the read counts of miRNA and tRNA were subjected to differential expression analysis using DESeq2 in R. The miRNAs and tRNAs were determined to be significantly changed if the adjusted *p*-values were below 0.05.

## 3. Results

### 3.1. Characterization of Rat Primary Schwann Cells and the Derived EVs

**Figure 1:**
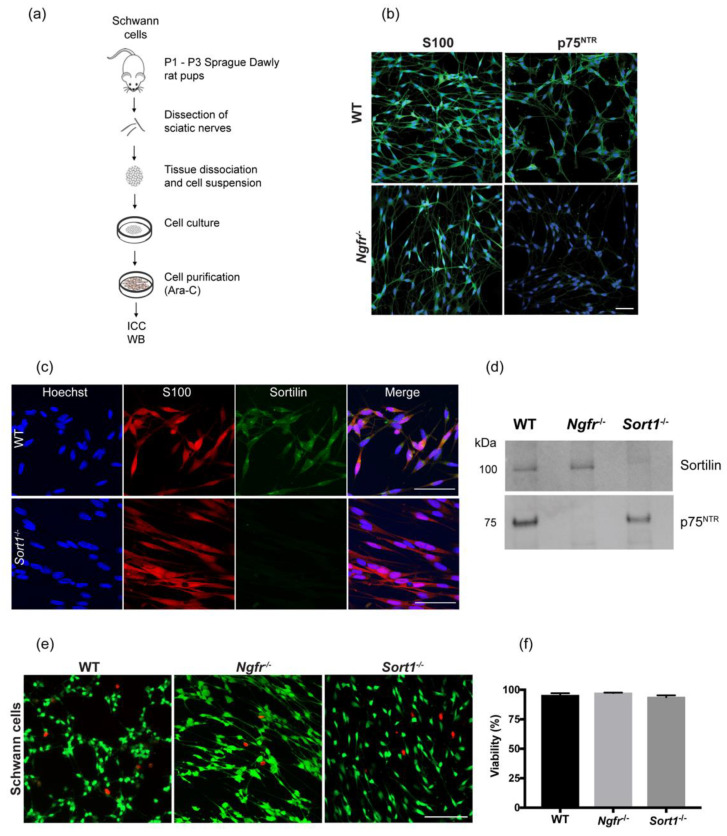
Primary Schwann cell (SC) characterization. (**a**) Schematic representation of the cell isolation strategy used. (**b**) Depiction of SC primary culture with immunocytochemistry against S100 and p75^NTR^ (both in green) in primary SCs from wild-type (WT) or *Ngfr*^−/−^ neonatal rats. Images show lack of p75^NTR^ staining in *Ngfr*^−/−^ derived cells. Nuclei are labeled in blue with Hoechst. Scale bar 50 μm. (**c**) Double immunocytochemistry between sortilin (red) and S100 (green) for both WT and *Sort1*^−/−^ derived primary SCs. Hoechst was used to label the cell nuclei (blue). Scale bar 50 μm. (**d**) Representative immunoblot for sortilin and p75^NTR^ from primary SC lysates, validating sortilin or p75^NTR^ protein absence in *Sort1*^−/−^ or *Ngfr*^−/−^ primary SCs, respectively. (**e**) Live and death assay where viable cells were identified in SC cultures by Calcein green fluorescence, while dead cells were labeled red by ethidium homodimer-1. Scale bar 100 μm (**f**) Quantification shows that viability of *Ngfr*^−/−^ or *Sort1*^−/−^ SCs is similar to that of WT SCs.

Sciatic nerves from P1–P3 neonatal WT, *Ngfr*^−/−^ and *Sort1*^−/−^ rat pups were used for primary SC cultures (Figure 1a) with >90% purity as demonstrated by staining with the SC marker S100 (Figure 1b, left column and Figure 1c). Immunocytochemistry and Western blot analysis validate deletion of p75^NTR^ and sortilin in primary SCs derived from *Ngfr*^−/−^ or *Sort1*^−/−^ rats, respectively (Figure 1b–d). As p75^NTR^ has been reported to regulate cell death/survival, we next investigated if lack of p75^NTR^ or the interacting partner sortilin could affect the viability of SCs in vitro. Labelling viable SCs with green fluorescence by Calcein (green) and dead cells red by ethidium homodimer-1 demonstrated that the percentage of viable *Ngfr*^−/−^ or *Sort1*^−/−^ SCs were similar to WT SCs (Figure 1e,f).

**Figure 2:**
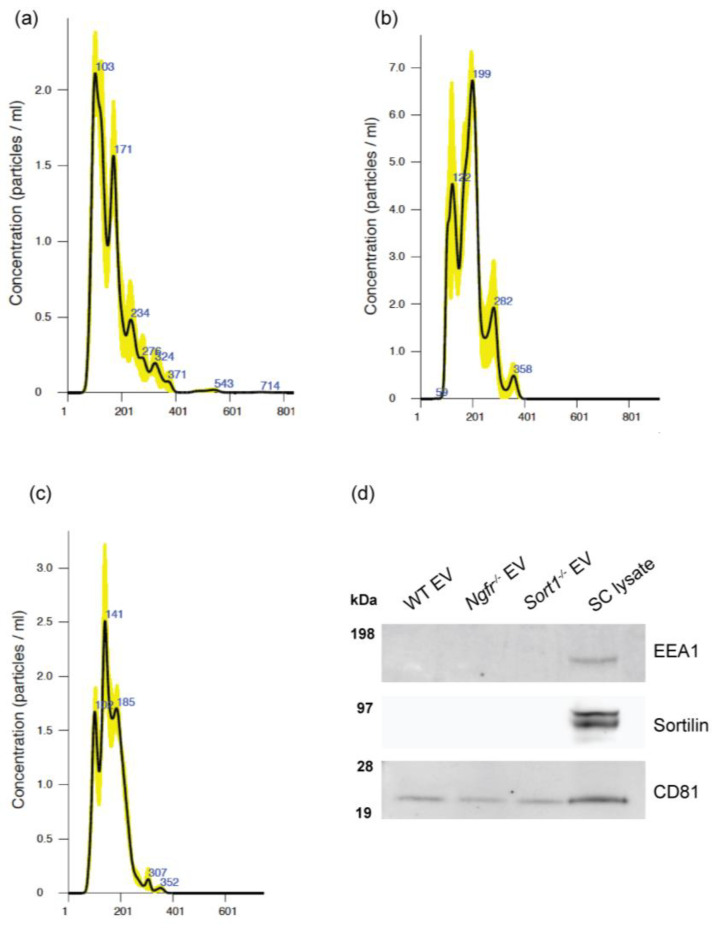
Size distribution of vesicles secreted by the primary Schwann cells (SCs). Representative plots with nanoparticle tracking analysis results of extracellular vesicles (EVs) collected from 3 × T175 flasks from (**a**) WT, (**b**) *Sort1*^−/−^ and (**c**) *Ngfr*^−/−^ derived rat primary SCs. (**d**) Immunoblot showing CD81 expression in both the EV pellets and SC lysate, while EEA1 and sortilin were only found present in the SC lysate.

Subsequently, EVs were purified from cell culture supernatants by differential ultracentrifugation and nanoparticle tracking analysis was used to measure size distribution of EVs in each sample. The EVs ranged in size between 50 nm and 400 nm, with similar size and distribution (Figure 2a–c) when derived from WT, *Sort1*^−/−^ or *Ngfr*^−/−^ Schwann cells. This indicates that both exosomes (30 nm to 150 nm in diameter) and microvesicles (ranging from 100 nm to 1 μm in diameter) are present in our samples [24]. In addition, analysis of the WT SC-derived EV pellet by LC–MS/MS confirmed expression of transmembrane and cytosolic proteins used as exosome markers, such as CD81, CD9 and TSG101; while several cell related proteins were absent from the dataset (Table S1). This finding was further corroborated with Western blot analysis demonstrating CD81 expression in the EV pellets and cell lysates, while EEA1 (as an early endosome marker) was only observed in the SC-lysate (Figure 2d). The lack of expression of this cell organelle marker demonstrates that cell/organelle contamination in our samples is residual. In addition, sortilin, which primarily locates in the trans-golgi network and intracellular vesicles, was not found in these SC-derived EVs.

Taken together, these results demonstrate that all WT, *Sort1*^−/−^ and *Ngfr*^−/−^ rodent primary SCs in culture can release EVs.

### 3.2. Small RNA Profile Identification in EVs Secreted from WT Rat Primary Schwann Cells

**Figure 3:**
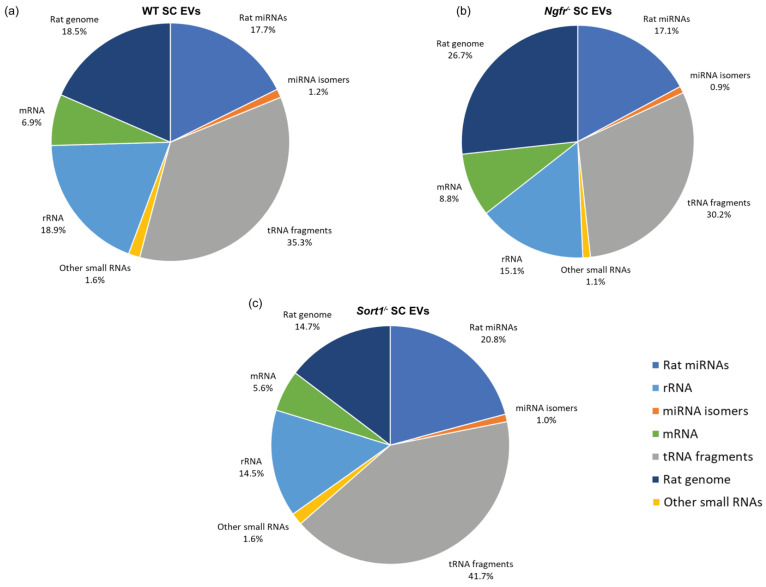
The read mapping distribution for the three types of Schwann cells (SCs) analyzed: (**a**) WT, (**b**) *Ngfr*^−/−^ and (**c**) *Sort1*^−/−^ SCs. The tRNA category includes all types of tRNA fragments.

Annotation of the small RNA expression pattern of the WT SC-derived EVs after sequencing demonstrated that most of the genome mapping reads were tRNA derived, followed by miRNA and rRNA, with only about 7% of the mapped reads relating to mRNA (Figure 3a)—a pattern that was similarly observed for EVs deriving from *Ngfr*^−/−^ and *Sort1*^−/−^ Schwann cells (Figure 3b,c).

**Figure 4:**
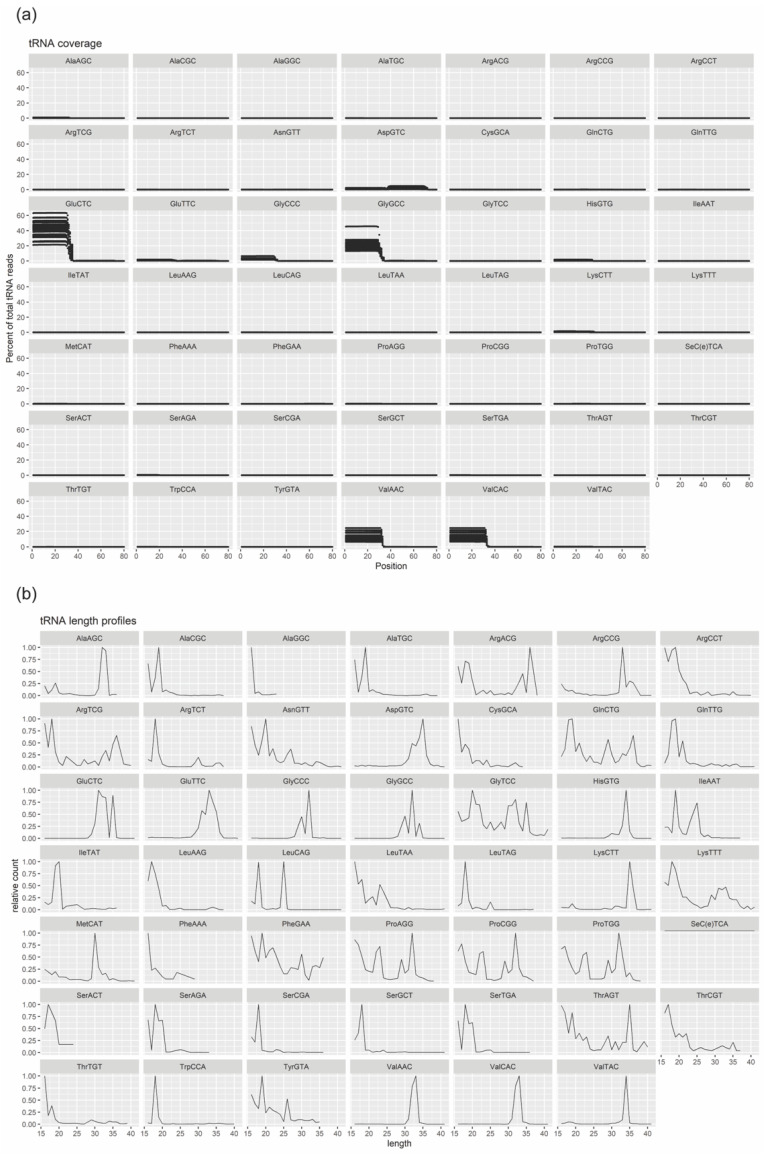
Characterization of WT SC-derived EV cargo in terms of tRNA fragments (tRFs) and halves (tiRNA). (**a**) Reads compiled on the individual tRNA maps. (**b**) The length of reads mapping to the various tRNAs.

Based on this, we next performed an in-depth analysis of the small RNAs with the largest number of sequenced reads which were tRNA derived RNA (mainly 20–22 nt tRNA-derived fragments (tRFs) and 33–35 nt tRNA halves (tiRNAs), and miRNA. Percentage and length of reads, mapping the various tRNA, were determined in the WT SC EVs (Figure 4a,b). The most highly expressed tRNAs were GluCTC, GlyGCC, ValAAC and ValCAC and they are all predominantly processed into 5′ tRNA halves. Only the lowest expressed AspGTC produced predominantly 3′ tRNA.

The 20 most highly expressed miRNAs in WT SC EVs (Table 1) were chosen for target prediction and pathway analysis.

**Figure 5:**
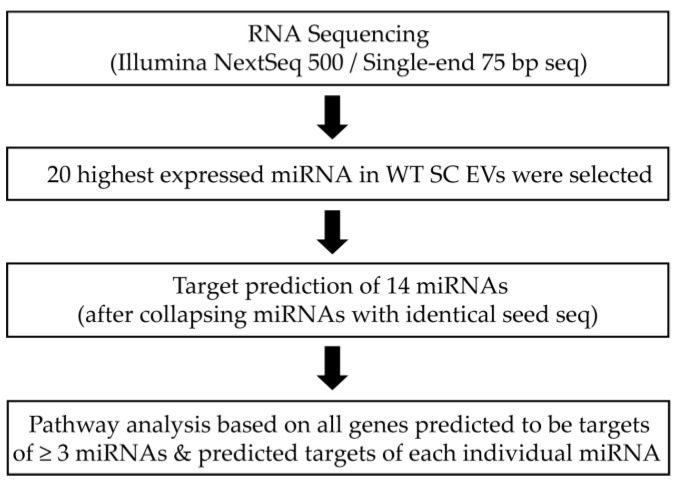
Pipeline for the miRNA analysis in EVs derived from WT SCs.

Since target predictions are focused on the seed sequence, miRNAs with identical seed sequences, such as let-7 family and miR-125 family, were ascribed the same target genes and collapsed into single entries (let-7-5p and miR-125-5p). As a result, target prediction using the algorithms miRWalk, miRanda and Targetscan was performed for 14 miRNAs in total. Only targets detected by all three algorithms were chosen (Figure 5).

**Figure 6:**
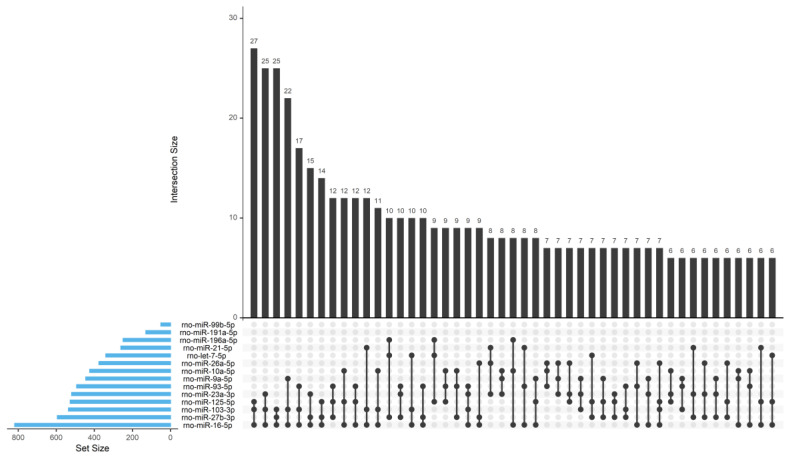
Characterization of WT SC-derived EV cargo in terms of miRNA content. Upset plot that shows the instances where more than 2 miRNAs target more than 5 genes. The plot is made using the R package “UpSetR”. The blue bars on the left side of the plot show the number of targets predicted for each miRNA. The right-side bar plot show the number of genes targeted by only a single miRNA (single black dot in the lower right panel) or multiple miRNAs (multiple linked dots in the lower right panel).

Most genes were predicted to be a target of a single miRNA, but a substantial number of genes were also predicted to be targeted by multiple miRNAs, as shown in Table 2 and Figure 6.

**Figure 7:**
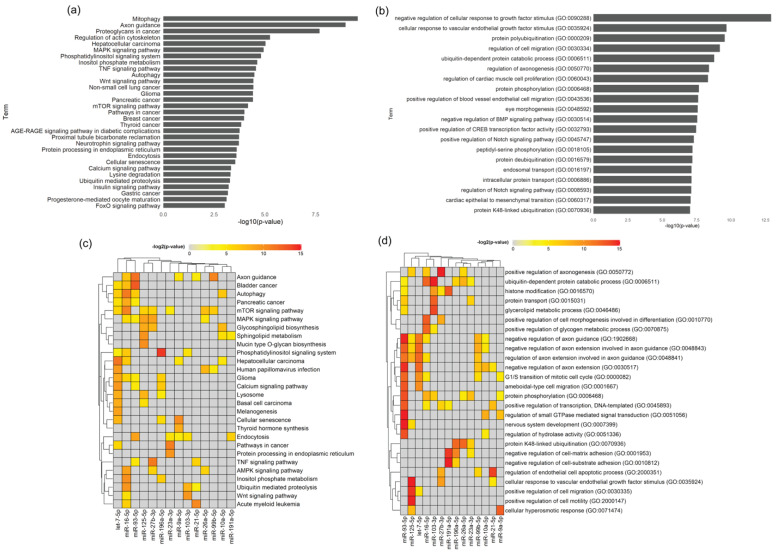
WT Schwann cell EV Pathway and Process analysis. (**a**) Pathway analysis for all genes predicted to be targets of 3 or more miRNAs. Hits from KEGG pathways with *p*-value < 0.05. Cancer hits were removed. (**b**) Pathway analysis for all genes predicted to be targets of 3 or more miRNAs. The top 20 hits from Gene Ontology Biological Process. (**c**) Pathway analysis for predicted targets of each individual miRNA. The 30 most significant hits from KEGG pathways are shown. The heatmap shows the level of significance—red being the most significant, grey meaning not significant. (**d**) Process analysis for predicted targets of each individual miRNA. The 30 most significant hits from Gene Ontology Biological Process are shown. The heatmap shows the level of significance—red being the most significant and grey representing non significance.

The predicted target genes were used to perform pathway analysis using the R package “enrichR”. Using this package, enrichment of two standard pathway/ontology datasets: KEGG Pathway and Gene Ontology Biological Process, demonstrated that genes predicted to be targets of three or more miRNAs are related with mitophagy, axon guidance, regulation of actin cytoskeleton, inositol metabolism, autophagy and several cellular signaling pathways (Figure 7a) linked to cell migration, proliferation, axonogenesis, protein phosphorylation and transport (Figure 7b). Additionally, hits from KEGG Pathways and Gene Ontology Biological Process on the predicted targets of each individual miRNA further highlighted the importance of Schwann-cell-derived EVs for axonal homeostasis. In this regard, particularly miR-93-5p, miR-99b-5p and let-7-5p were shown to be the most significant miRNA for axon guidance (Figure 7c,d), with miR-93-5p and let-7-5p being also involved in axon extension and miRNA-27b-3p with roles in axonogenesis (Figure 7d). In addition, miR-93-5p regulates genes strongly linked with nervous system development and thus, overall, in the present dataset, this miRNA seems to be the most important for regulation of axonal related processes. The impact of SC signaling through EVs upon other SCs or non-neuronal cell types was also evidenced by the presence of miRNAs important for regulation of the endothelial cell apoptotic process (specially by miRNA-125-5p), migration, motility (particularly, miRNA-21-5p) (Figure 7d) and several signaling pathways. From all the KEGG signaling pathways, MAPK and mTOR are the ones connected to a higher number of miRNAs (Figure 7c). Interestingly, signaling pathways linked with diabetic complications, such as insulin or the advance glycation end products (AGE) binding to their receptor RAGE signaling (Figure 7a) and lipid metabolism (Figure 7c,d) were annotated, which might be relevant in the context of the contribution of Schwannopathy for the pathogenesis of diabetic neuropathy [25].

### 3.3. Several Genes in the Axon Guidance Signaling Pathway Are Predicted Targets by Identified miRNA

Axon guidance is one of the signaling pathways identified by highest significance (Table 3). Therefore, we next analyzed genes from this pathway targeted by five of the most expressed miRNAs present in SC-derived EVs. Among the 180 distinct genes belonging to the axon guidance KEGG signaling pathway, 29 genes are predicted to be targeted and regulated by identified miRNAs (Table 4), which included receptors, ligands and activated receptors. It is noticeable that a large number of these target genes belong to the same gene families such as Ephrins, Semaphorins, Netrins, and Slits, with Semaphorins comprising the big majority (Table 4).

### 3.4. Differential Expression of Small RNAs in Schwann Cells Lacking p75^NTR^ or Sortilin

**Figure 8:**
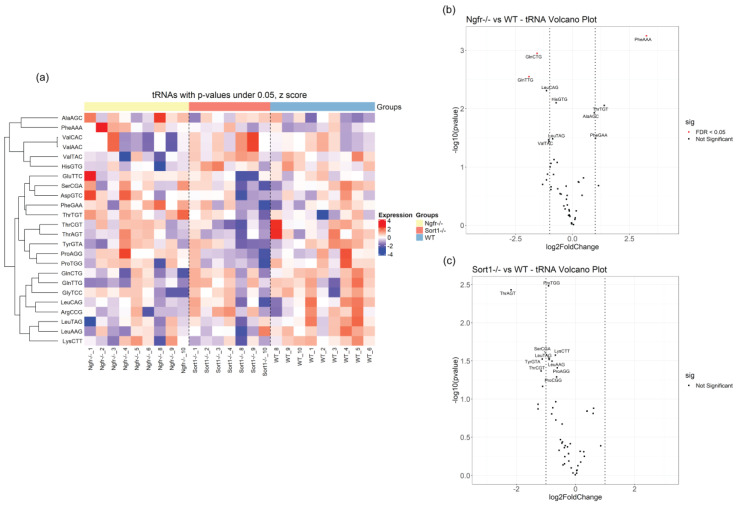
Differentially expressed tRNAs in EVs derived from *Ngfr*^−/−^ and *Sort1*^−/−^ compared with EVs from WT Schwann cells. (**a**) Heatmap covering the most significantly changing tRNAs, with *p*-values (not multiple-testing corrected) below 0.05 in one or more of the pairwise comparisons. Top annotation shows *Sort1*^−/−^ in orange, *Ngfr*^−/−^ in yellow and control WT as blue. Shown are z scores of log2-transformed RPM values. (**b**) Volcano plot for EV tRNAs from *Ngfr*^−/−^ versus WT comparison and (**c**) Volcano plot for EV tRNAs from *Sort1*^−/−^ versus WT. Plotted are –log10(*p*-values) vs. −log2(fold change). Red means < 0.05 adjusted *p*-value. Vertical lines indicate +/− 1 log2(fold change). The names for the 10 most significant tRNA are indicated.

As tRNAs accounted for the larger percentage of mapped reads (Figure 3a–c), we conducted differential expression analysis of tRNAs, using DESeq2 in R. The most significantly expressed tRNAs are depicted in the heatmap of Figure 8a. The number of differentially expressed tRNAs with adjusted *p*-values below 0.05 or *p*-values (without adjusting for multiple testing) below 0.05 can be seen in Table 5. Although tRNA constitutes the highest population of small RNA identified in the primary SC EVs, only a few of these are differentially regulated in *Ngfr*^−/−^ vs. WT after multiple testing correction, namely, PheAAA, GlnCTG and GlnTTG (Figure 8b). The tRNA length profile (Figure 4b) showed that these three differentially expressed tRNA fragments predominantly include the shorter 5′-end tRFs in these samples. When removing expression of *Sort1*, no significant differences were observed, as illustrated in the volcano plot on Figure 8c.

**Figure 9:**
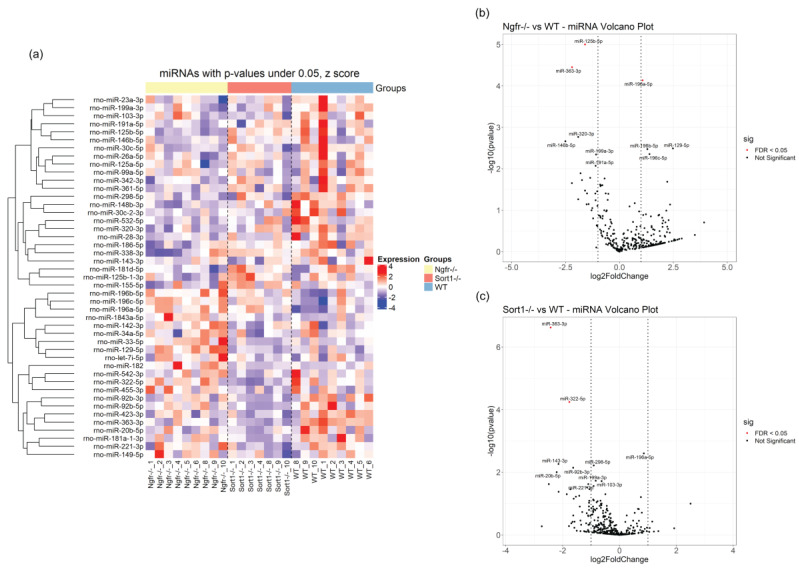
Differentially expressed miRNAs in EVs derived from *Ngfr*^−/−^ and *Sort1*^−/−^ compared with EVs from WT Schwann cells (SCs). (**a**) The 45 most significantly differentially expressed miRNAs (not multiple-testing corrected) are shown as a heatmap covering all samples from WT, *Ngfr*^−/−^ and *Sort1*^−/−^ SCs. Top annotation shows *Sort1*^−/−^ in orange, *Ngfr*^−/−^ in yellow and control WT as blue. Shown are z scores of log2-transformed RPM values. (**b**) Volcano plot for EV miRNAs from *Ngfr*^−/−^ versus WT comparison and (**c**) Volcano plot for EV miRNAs from *Sort1*^−/−^ versus WT. Plotted are –log10(*p*-values) vs. −log2(fold change). Red means < 0.05 adjusted *p*-value. Vertical lines indicate +/− 1 log2(fold change). The names for the 10 most significant miRNA are indicated.

All miRNAs with *p*-values below 0.05 are shown on the heatmap in Figure 9a. In general, few miRNAs differ upon deletion of p75^NTR^ (Figure 9b) or sortilin (Figure 9c); however, these were highly affected with very significant adjusted *p*-values (Table 6 and Table 7), suggestive of important regulatory gene function. Most of the differentially expressed miRNAs were downregulated (Figure 9b,c), with only miRNA-196a-5p found upregulated (Figure 9b). Three downregulated miRNAs were also found when comparing EVs derived from *Sort1*^−/−^ versus *Ngfr*^−/−^ primary Schwann cells (Table 7), indicating differential impact regarding Schwann cell EVs cargo.

### 3.5. Target Genes and Target Pathways for the Differentially Expressed miRNAs Identified in EVs from Primary Schwann Cells Lacking p75^NTR^ or Sortilin

The miRNAs previously found to have adjusted *p*-values below 0.25 when comparing *Ngfr*^−/−^ vs. WT (9 miRNAs) and *Sort1*^−/−^ vs. WT (2 miRNAs) were chosen for target prediction and pathway analysis (Table 8).

**Figure 10:**
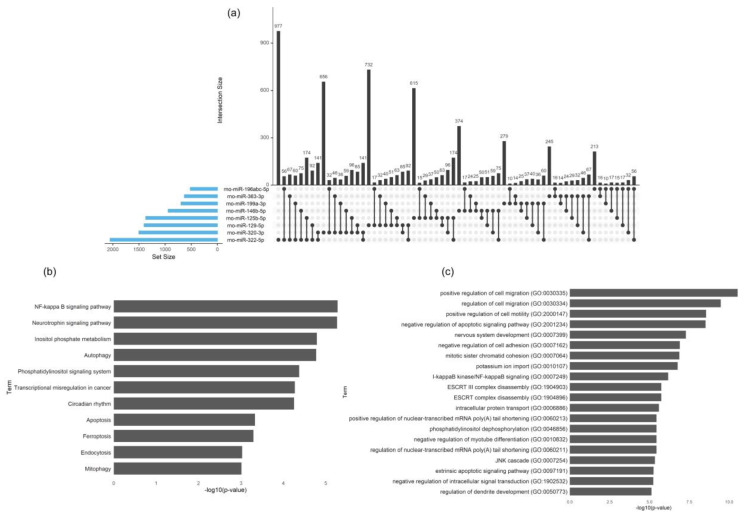
Transgenic Schwann cell EV cargo characterization (lacking p75^NTR^ or sortilin expression). (**a**) UpSet plot showing the number of predicted target genes found to be targeted by individual miRNAs or by multiple miRNAs. The plot is made using the R package “UpSetR”. The blue bars on the left side of the plot show the number of targets predicted for each miRNA. The right-side bar plot shows the number of genes targeted by only a single miRNA (single black dot in the lower right panel) or multiple miRNAs (multiple linked dots in the lower right panel). (**b**) Pathway analysis for all genes predicted to be targets of 3 or more miRNAs. All hits from KEGG pathways with *p*-value < 0.05. (**c**) Pathway analysis for all genes predicted to be targets of 3 or more miRNAs. The top 20 hits from Gene Ontology Biological Process.

miR-363-3p was found downregulated in both comparisons. miR-199a-3p, miR-199b-3p and miR-199c-3p have identical seed sequences, causing target prediction algorithms to ascribe them the same targets and collapsing them into the term miR-199abc-3p. Altogether, this resulted in eight miRNAs to be analyzed for target prediction using multiple algorithms simultaneously. The prediction algorithms used were miRWalk, miRanda and Targetscan and only targets detected by all three algorithms were chosen. The number of genes targeted by each of the miRNAs is shown in Figure 10a. Most genes were only predicted to be target of a single miRNA (*n* = 4091), but a substantial number of genes were also predicted to be targeted by multiple miRNAs (Figure 10a). To identify pathways enriched with these genes, a Gene Ontology Biological Process and KEGG pathway analysis was performed. Figure 10b,c illustrate the name of the pathways and biological processes for all genes predicted to be targets of three or more miRNAs.

**Figure 11:**
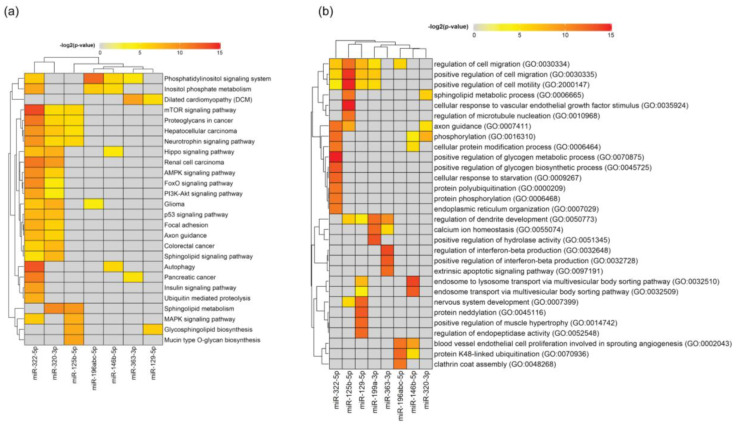
Pathway analysis for *Ngfr*^−/−^ and *Sort1*^−/−^ Schwann cell EV cargo. (**a**) Pathway analysis for predicted targets of each individual miRNA. The 30 most significant hits from KEGG pathways are shown. The heatmap shows the level of significance—red being most significant, grey means not significant. (**b**) Pathway analysis for predicted targets of each individual miRNA. The 30 most significant hits from Gene Ontology Biological Process are shown. The heatmap shows the level of significance—red being most significant, grey means not significant.

To illustrate the complexity of the possible interacting effects of the candidate miRNAs, we analyzed predicted target genes of each individual miRNA both for KEGG pathways (Figure 11a) and Gene Ontology Biological Process (Figure 11b). Excluding cancer pathways, the only pathway enriched with genes targeted simultaneously by the two differentially expressed miRNAs found in *Sort1*^−/−^ SC-derived EVs was the phosphatidylinositol signaling system (Figure 11a). Nevertheless, the most significant were pathways related with autophagy and the mTOR signaling, both coded by the miR-322-5p (Figure 11a). This miRNA seems to be also important for regulation of genes involved in axon guidance, neurotrophin and insulin signaling (Figure 11a,b), which is particularly interesting due to the known functions of sortilin for glucose homeostasis and neuronal viability in the periphery [18,26].

Similar to what happens with *Sort1*^−/−^ SC-derived EVs, also genes regulated simultaneously by two differentially expressed miRNAs in EVs from *Ngfr*^−/−^ primary Schwann cells, as compared to WT, belong to the phosphatidylinositol signaling system, which, in this case, also corresponds to the most significant pathway found (Figure 11a), indicating that EVs incorporate information for lipid signaling and membrane trafficking. Sphingolipid metabolism, NF-kB, MAPK signaling pathways, cell migration and the apoptotic signaling extrinsic pathway are some of the most significant pathways/processes targeted by miRNAs from *Ngfr*^−/−^ EVs (Figure 11a,b).

## 4. Discussion

Peripheral nerve roots, trunks and terminals are connected with SCs, the principal glial cells of the PNS. They derive from the neural crest cells and in their mature state SCs can be divided into myelinating SCs, which insulate axons with concentric layers of a lipid-rich myelin sheath, nonmyelinating SCs, that ensheath numerous small caliber axons in organized structures classified as Remak bundles, and terminal or perisynaptic SCs, located at the neuromuscular junction [27,28]. Recently, new SC subtypes have been described, such as the nociceptive SCs in the skin [29] and the repair SCs, originating in the distal segment of an injured nerve to promote repair [30]. Something they all have in common is their critical role in maintaining axonal and neuronal homeostasis under normal physiological conditions by bidirectional SC-axon communication and by interacting with the extracellular matrix and other cell types [31]. This intercell communication is crucial for SC-mediated myelination [32], survival [33], as well as for directing their migration patterns [34,35] or activating dedifferentiation after a PNS injury [36]; but also for axonal support and PNS plasticity [10]. Several are the proposed signaling transmitting mechanisms, however, in the last decade, axo-glial communication through the release of exosomes has gained considerable insights. In vitro released SC-derived exosomes were shown to be internalized by sensory neurons, stimulating axonal regeneration both in vitro and in vivo [9], which opened a new dimension in our understanding of glia-to-axon transfer of molecular signals. Nevertheless, up to date, little is known regarding SC exosome cargo. Very recently, the proteomic profile of rat primary SC-derived exosomes was characterized [37]. Similar to those findings, with regard to enriched KEGG pathways within the exosome proteins, also in the present study we found that the regulation of actin cytoskeleton, axon guidance, PI3K-Akt and neurotrophin signaling pathways to be amongst the most enriched pathways regulated by the identified miRNA in SC-derived EVs. Hence, this suggests that miRNA present among the SC EV cargo may regulate neurorestorative genes, proteins and pathways in the receiving cell, opening a new avenue for potential therapeutic applications in regions of the nervous system with poor regenerative capabilities, such as the CNS [38]. When compared with a recent study [39], a lower percentage of miRNA and a higher percentage of tRNA in SC-derived EVs were here identified, which might be related with different culture methodologies, environmental factors or distinctive parameters/cut-offs involved in the analysis. The identified miRNA cargo of the Schwann-cell-derived EVs is involved in the basic biological pathways like endocytosis, mitophagy, autophagy and regulation of cytoskeleton, indicating that EVs can reprogram the phenotype and regulate the function of the recipient cells in the peripheral nerve. This EV-mediated crosstalk may be critical to maintain the homeostasis of the glial–axon syncytium.

Importantly, the axon–SC interlaced union during development and throughout life is crucial for the homeostasis of the PNS but also upon disease. Axon guidance, one of the main regulated pathways by miRNA present in SC-derived EVs found in this study, is involved in the formation of neuronal networks at the growth cone and is directed by several families of proteins including semaphorins, ephrins, slits, netrins and their corresponding receptors [40]. Recent reports implicated this pathway and some of these proteins as being involved in the pathogenesis of diabetic retinopathy and nephropathy [41,42], as well as associated with circulating miRNA profiles found in plasma of hyperglycemic patients with type I diabetes [43]. Due to the important documented role of SCs for the development of diabetic neuropathy [25,44], it might be worth exploring if axon guidance related molecules are also involved in the development of this main diabetes complication. In fact, it was recently demonstrated that exosomes derived from high glucose-stimulated primary SCs suppress axonal growth in vitro and promote the development of diabetic peripheral neuropathy in the db/db mouse model [14]. In parallel with this, a positive therapeutic effect of exosomes derived from healthy SCs on the db/db type 2 diabetic peripheral neuropathy mouse line was documented [15], but with no assessment of exosome cargo. Therefore, results from the present study with disclosure of SC EV small RNA contents can help us gain a deeper understanding of potential therapeutic mechanisms for nerve regeneration and peripheral neuropathies.

In the neurotrophin signaling pathway, we found that 16 out of 121 assigned genes are predicted to be targeted by more than three regulated miRNAs. Neurotrophins play multiple roles in neural development, neurodegeneration, inflammation or neuropathic pain [45,46] with well-known impact upon the pathogenesis of peripheral neuropathies, such as diabetic neuropathy [47] or Charcot-Marie Tooth disease [48]. Neurotrophins can bind and activate two different classes of receptors, p75^NTR^ and the Trk family of tyrosine kinase receptors [49], with different signaling outcomes attained, involving cascades mediated by MAP kinase, PI-3K or Jun. In the PNS, the pleiotropic receptor p75^NTR^ is expressed by both SCs and sensory neurons facilitating cell death or survival, stimulating or restraining axonal growth and accelerating or weakening proliferation, depending on the cellular context and on binding to its co-receptors, such as sortilin [18,50]. Previously, it was found that p75^NTR^ was present as cargo in SC-secreted extracellular vesicles, being thus further suggested as a potential marker for SC-derived exosomes [9]. Clinically, both p75^NTR^ and sortilin have been implicated in the pathophysiology of some degenerative diseases, including Alzheimer’s disease [51,52]. In the PNS, the role of p75^NTR^ is still somewhat unresolved as it was recently shown that conditional ablation of this receptor in SCs does not impact remyelination after peripheral nerve crush injury [53]. Sortilin is also expressed by SCs in the periphery, but its involvement in nerve regeneration or PNS disease is yet to be determined. Therefore, we aimed at exploring the impact of deleting p75^NTR^ expression or its co-receptor sortilin on the release and small RNA content of primary SC-derived EVs. SCs lacking p75^NTR^ or sortilin were still able to produce and release EVs and, in contrast with what was observed for exosomes deriving from human lung carcinoma cells [23], SC-derived EVs do not seem to carry sortilin—a finding that highlights the specificity of EV cargo from cell to cell. Overall, few EV miRNAs were differentially expressed but with potential of targeting a dozen of pathways and hundreds or thousands of genes. Most of the enriched miRNAs found in EVs from the transgenic primary SCs had gene targets in the NF-kB and PI3K signaling pathways, suggesting that these EVs may incorporate more information to increase immune regulation and survival mechanisms in recipient cells. miR-125b-5p, found down-regulated in EVs from *Ngfr*^−/−^ SCs, was previously associated with IL-1β induced inflammation [54], which is very interesting due to the known relationship between IL-1 and p75^NTR^ [55,56]. Several studies highlighted different functions of sortilin in the immune system [57,58], but this receptor has also been linked to type 2 diabetes and obesity, cancer and cardiovascular pathologies. In this regard, miR-363-3p, that was here identified as downregulated in both transgenic derived SC EVs, has been linked to insulin resistance and post-stroke depressive behavior [59,60]. This, together with the reduced levels of miR-322-5p that was previously found important for targeting IGF-1 signaling [61], suggests a potential involvement of SC expression of sortilin in diabetes or diabetic complications. It was very recently demonstrated that SC p75^NTR^ deficiency amplifies diabetic neuropathy disease by over-activating immune-related pathways and an increase in lysosomal stress [62]. One hypothesis is that miR-196a and/or miR-125b-5p, here found enriched in *Ngfr*^−/−^ SC EVs, could potentially play a role in the observed diabetic phenotype in mice lacking SC p75^NTR^ expression as they were recently associated with type 1 diabetes [43] and in the regulation of body fat distribution [63].

The most abundant small RNA in SC-derived EVs in our study was tRNA-derived small RNAs, which is consistent to other studies using other cell types [64,65]. We found that tRFs derived from PheAAA, GlnCTG and GlnTTG were differentially expressed in the EVs derived from *Ngfr*^−/−^ SCs compared to WT EVs, while the sortilin knockdown did not result in any significant change of expression for tRNA fragments. It has been shown that tRFs can function as miRNAs by binding Argonaute proteins and repressing gene expression [66]. Some studies demonstrated the association between the abnormal expression of tRFs and neurological disorders, such as Parkinson’s disease [67], ischemia-reperfusion injury [68] and cerebellar neurodegeneration [69]. However, further investigation is needed to understand the function of the small RNAs derived from tRNAs in EVs secreted from SCs and the association with glial p75^NTR^.

The role of EVs for cell-to-cell communication has gained substantial attention in recent years. They seem to be crucial factors for glia-to-axon transfer of proteins and RNA, regulating this functional syncytium in health and disease. miRNA signatures show promise as future biomarkers for several disease states due to their cell type-specific expression patterns. Therefore, the present preliminary work contributes especially to the knowledge of SC miRNA expression patterns to be used in future research targeting physiology in health and disease. Further complementary functional studies will be necessary to understand specific biological mechanisms related with particular miRNA targets in the context of PNS homeostasis.

## Figures and Tables

**Table 1:** The 20 miRNAs highest expressed in EVs derived from WT rat primary Schwann cells. RPM stands for reads per million mapped reads.

miRNA	Mature Sequence	Seed	Seed Family	Mean Expression (RPM)
rno-let-7a-5p	UGAGGUAGUAGGUUGUAUAGUU	GAGGUAG	let-7-5p	72,693
rno-let-7b-5p	UGAGGUAGUAGGUUGUGUGGUU	GAGGUAG	let-7-5p	145,454
rno-let-7c-5p	UGAGGUAGUAGGUUGUAUGGUU	GAGGUAG	let-7-5p	167,214
rno-let-7e-5p	UGAGGUAGGAGGUUGUAUAGUU	GAGGUAG	let-7-5p	19,762
rno-let-7f-5p	UGAGGUAGUAGAUUGUAUAGUU	GAGGUAG	let-7-5p	90,702
rno-let-7i-5p	UGAGGUAGUAGUUUGUGCUGUU	GAGGUAG	let-7-5p	143,004
rno-miR-103-3p	AGCAGCAUUGUACAGGGCUAUGA	GCAGCAU	miR-103-3p	5290
rno-miR-10a-5p	UACCCUGUAGAUCCGAAUUUGUG	ACCCUGU	miR-10a-5p	8054
rno-miR-125a-5p	UCCCUGAGACCCUUUAACCUGUGA	CCCUGAG	miR-125-5p	17,510
rno-miR-125b-5p	UCCCUGAGACCCUAACUUGUGA	CCCUGAG	miR-125-5p	16,168
rno-miR-16-5p	UAGCAGCACGUAAAUAUUGGCG	AGCAGCA	miR-16-5p	61,196
rno-miR-191a-5p	CAACGGAAUCCCAAAAGCAGCUG	AACGGAA	miR-191a-5p	4979
rno-miR-196a-5p	UAGGUAGUUUCAUGUUGUUGGG	AGGUAGU	miR-196a-5p	8995
rno-miR-21-5p	UAGCUUAUCAGACUGAUGUUGA	AGCUUAU	miR-21-5p	79,396
rno-miR-23a-3p	AUCACAUUGCCAGGGAUUUCC	UCACAUU	miR-23a-3p	5924
rno-miR-26a-5p	UUCAAGUAAUCCAGGAUAGGCU	UCAAGUA	miR-26a-5p	8792
rno-miR-27b-3p	UUCACAGUGGCUAAGUUCUGC	UCACAGU	miR-27b-3p	7431
rno-miR-93-5p	CAAAGUGCUGUUCGUGCAGGUAG	AAAGUGC	miR-93-5p	4785
rno-miR-99b-5p	CACCCGUAGAACCGACCUUGCG	ACCCGUA	miR-99b-5p	6245
rno-miR-9a-5p	UCUUUGGUUAUCUAGCUGUAUGA	CUUUGGU	miR-9a-5p	13,541

**Table 2:** Table showing the number of predicted target genes found to be targeted by individual miRNAs or by multiple miRNAs.

miRNAs Targeting	1	2	3	4	5	6	7	8	9	10
Number of target genes	4072	1947	881	394	157	67	32	10	3	2

**Table 3:** KEGG pathways enriched by genes targeted by the 14 candidate miRNAs (cancer pathways are not included).

miRNA(s)	KEGG Pathways	Total	Targeted	*p*	*P_adjust_*
*n* =14	Mitophagy	63	15	6.92 × 10^−5^	0.02095 *
*n* =14	Axon guidance	180	29	1.25 × 10^−4^	0.01896 *
*n* =13	Regulation of actin cytoskeleton	217	28	5.19 × 10^−3^	0.39371
*n* =13	MAPK signaling pathway	294	35	7.12 × 10^−3^	0.35967
*n* =14	Phosphatidylinositol signaling system	98	15	8.12 × 10^−3^	0.35154
*n* =14	Inositol phosphate metabolism	73	12	9.76 × 10^−3^	0.36990
*n* =13	TNF signaling pathway	110	16	0.01036	0.34905
*n* =13	Autophagy	130	18	0.01122	0.34022
*n* =13	Wnt signaling pathway	160	21	0.01180	0.32508
*n* =14	mTOR signaling pathway	154	20	0.01532	0.30952
*n* =12	AGE-RAGE signaling pathway in diabetic complications	101	14	0.02327	0.37114
*n* =13	Neurotrophin signaling pathway	121	16	0.02408	0.34750
*n* =13	Protein processing in endoplasmic reticulum	163	20	0.02671	0.36798
*n* =14	Endocytosis	269	30	0.02725	0.35904
*n* =12	Cellular senescence	185	22	0.02868	0.36211
*n* =13	Calcium signaling pathway	189	22	0.03532	0.42808
*n* =12	Lysine degradation	59	9	0.03634	0.42351
*n* =13	Ubiquitin mediated proteolysis	138	17	0.03756	0.42161
*n* =14	Insulin signaling pathway	139	17	0.03984	0.43120
*n* =13	Progesterone-mediated oocyte maturation	90	12	0.04402	0.44468
*n* =12	FoxO signaling pathway	132	16	0.04835	0.472584

* Pathways with strongest statistical support for enrichment, with genes targeted by 14 out of 14 candidate miRNAs.

**Table 4:** List of genes in the axon guidance pathway targeted by five candidate miRNAs.

miRNA(s)	Ephrins	Semaphorins	Netrins	Slits	Other Genes
miR-93-5p	EPHA5	SEMA5A	UNC5C	SLIT2	CXCL12
miR-16-5p	EPHA4	SEMA3D	NTNG1	ROBO2	LIMK1
miR-21-5p		SEMA3G			LIMK2
miR-99b-5p		SEMA6D			NFATC3
miR-9a-5p		SEMA4B			CAMK2B
		SEMA4C			ROCK2
		PLXNA2			NRAS
		PLXNC1			ABLIM1
					PARD6B
					CFL2
					PLCG1
					NEO1
					SSH2
					RGMA
					PIK3CA

**Table 5:** Numbers of differentially expressed tRNAs with *p*_adjust_ values below 0.05 or *p* values (without adjusting for multiple testing) below 0.05.

Comparisons	*p* < 0.05	P_adjust_ < 0.05
*Ngfr*^−/−^ vs. WT	11	3
*Sort1*^−/−^ vs. WT	9	0
*Ngfr*^−/−^ vs. *Sort1*^−/−^	14	0

**Table 6:** Numbers of differentially expressed miRNAs with *p* adjust values below 0.05 or *p* values (without adjusting for multiple testing) below 0.05.

Comparisons	*p* < 0.05	P_adjust_ < 0.05
*Ngfr*^−/−^ vs. WT	26	3
*Sort1*^−/−^ vs. WT	16	2
*Ngfr*^−/−^ vs. *Sort1*^−/−^	16	3

**Table 7:** The most differentially expressed miRNAs in EVs from rat primary Schwann cells lacking p75^NTR^ or sortilin.

Comparison	miRNA	Log2ratio	Fold Change	*p*	*P_adjust_*
*Ngfr*^−/−^ vs. WT	miR-125b-5p	−1.598058994	3.031	1.00 × 10^−5^	0.003998687
	miR-363-3p	−2.203581831	4.610	3.55 × 10^−5^	0.007104917
	miR-196a-5p	1.073004433	2.104	7.33 × 10^−5^	0.009770739
*Sort1*^−/−^ vs. WT	miR-363-3p	−2.423798775	5.366	2.38 × 10^−7^	9.53 × 10^−5^
	miR-322-5p	−1.761922373	3.391	5.72 × 10^−5^	0.011432346
*Ngfr*^−/−^ vs. *Sort1*^−/−^	miR-322-5p	−1.930509951	3.812	5.68 × 10^−5^	0.016886126
	miR-129-5p	−3.790721064	13.840	1.24 × 10^−4^	0.016886126
	miR-142-3p	−1.612982475	3.059	1.26 × 10^−3^	0.016886126

**Table 8:** List of miRNAs selected for target prediction and pathway analysis.

miRNA	*p* Value	*P*_adj_ Value
	***Ngfr*^−/−^ vs. WT**	
miR-125b-5p	1.00 × 10^−5^	0.003998687
miR-363-3p	3.55 × 10^−5^	0.007104917
miR-196a-5p	7.33 × 10^−5^	0.009770739
miR-320-3p	0.00171393	0.171392973
miR-146b-5p	0.00217757	0.174205628
miR-129-5p	0.003234721	0.192012662
miR-196b-5p	0.003360222	0.192012662
miR-196c-5p	0.004418208	0.201005403
miR-199a-3p	0.004522622	0.201005403
	***Sort1*^−/−^ vs. WT**	
miR-363-3p	2.38 × 10^−7^	9.53 × 10^−5^
miR-322-5p	5.72 × 10^−5^	0.011432346

## Supplementary Materials

The following are available online at https://www.mdpi.com/2227-9059/8/11/450/s1.
